# Impact of the TRPV2 Inhibitor on Advanced Heart Failure in Patients with Muscular Dystrophy: Exploratory Study of Biomarkers Related to the Efficacy of Tranilast

**DOI:** 10.3390/ijms24032167

**Published:** 2023-01-21

**Authors:** Chisato Takahashi, Mariko Oishi, Yuko Iwata, Keiko Maekawa, Tsuyoshi Matsumura

**Affiliations:** 1 Department of Analytical Chemistry, Faculty of Pharmaceutical Sciences, Doshisha Women’s College of Liberal Arts, Kyotanabe 610-0395, Kyoto, Japan; 2 Department of Cardiac Physiology, National Cerebral and Cardiovascular Center Research Institute, 6-1 Kishibe-Shimmachi, Suita 564-8565, Osaka, Japan; 3 Department of Neurology, National Hospital Organization Osaka Toneyama Medical Center, 5-1-1 Toneyama, Toyonaka 560-8551, Osaka, Japan

**Keywords:** muscular dystrophy, tranilast, transient receptor potential cation channel subfamily V member 2, heart failure, brain natriuretic peptide, metabolomics, oxidative fatty acids

## Abstract

Cardiomyopathy is the leading cause of death in patients with muscular dystrophy (MD). Tranilast, a widely used anti-allergic drug, has displayed inhibitory activity against the transient receptor potential cation channel subfamily V member 2 and improved cardiac function in MD patients. To identify urinary biomarkers that assess improved cardiac function after tranilast administration, we performed a urinary metabolomic study focused on oxidative fatty acids. Accompanying the clinical trial of tranilast, urine specimens were collected over 24 weeks from MD patients with advanced heart failure. Urinary levels of tetranor-PGDM (tetranor-prostaglandin D metabolite), a metabolite of prostaglandin D_2_, significantly decreased 12 weeks after tranilast administration and were correlated with BNP. These results suggest that prostaglandin-mediated inflammation, which increases with the pathological progression of heart failure in MD patients, was attenuated. Urinary prostaglandin E_3_ (PGE_3_) levels significantly increased 4 weeks after tranilast administration. There were positive correlations between the urinary levels of PGE_3_ and 8-hydroxy-2′-deoxyguanosine, an oxidative stress marker. High PGE_3_ levels may have a protective effect against cardiomyopathy in MD patients with high oxidative stress. Although further validation studies are necessary, urinary tetranor-PGDM and PGE_3_ levels may help the current understanding of the extent of advanced heart failure in patients with MD after tranilast administration.

## 1. Introduction

The transient receptor potential cation channel subfamily V member 2 (TRPV2) is a stretch-sensitive Ca^2+^ channel. It is normally localized in intracellular membrane compartments but translocates to the cytoplasmic membrane in damaged myocytes or cardiomyocytes and enhances the influx of Ca^2+^, which triggers the cell damage process [1]. Overexpression of TRPV2 at the sarcolemma was observed in the skeletal muscle and cardiomyocytes of patients with muscular dystrophy (MD) and animal models of MD such as mdx mice and BIO 14.6 hamsters [1,2]. Therefore, inhibition of TRPV2 is expected to suppress myocardial degeneration by a mechanism that differs from that of conventional cardioprotective agents such as angiotensin-converting enzyme inhibitors (ACEIs), angiotensin II receptor blockers (ARBs), and beta-blockers. Indeed, TRPV2 inhibition using mutant TRPV2 and Ca^2+^ handling agents was effective against heart failure [3] and motor function [4,5] in animal models of MD. We have already demonstrated that tranilast, a widely used anti-allergic drug, displays TRPV2-inhibitory activity and has beneficial effects in BIO14.6 hamsters [2] and mdx mice [6]. These results suggest that tranilast may be a useful therapeutic agent for patients with MD.

Currently, heart failure is the major cause of death in MD patients, including those with Duchenne muscular dystrophy (DMD) [7]. Heart failure progresses chronically and often concurrently with respiratory failure. Although novel therapies such as exon skipping are being developed for DMD, they are unlikely to affect myocardiopathy [8]. This raises the concern that improvements in motor dysfunction with novel therapies might increase the risk of cardiac burden. Therefore, there is an urgent need to develop therapies aimed at improving cardiac function. In addition, to assess cardiac function in patients with MD, multiple markers and serial evaluations are needed because magnetic resonance imaging and echocardiography are difficult to apply in many cases due to their pathological conditions. Brain natriuretic peptide (BNP) level is a standard cardiac function marker that is highly correlated with heart failure severity and prognosis. However, BNP level frequently remains low, even in the advanced stages of heart failure, because factors other than myocardial degeneration, such as instability of general conditions, volume load, vascular resistance, and blood pressure, also affect its levels.

Under these circumstances, we conducted a pilot study and multicenter clinical trial to assess the safety and efficacy of tranilast in MD patients with cardiomyopathy [3,9,10]. In the pilot study, reduced BNP levels were observed in two participating patients with severe cardiac dysfunction after 3 months of continuous administration of tranilast at 300 mg/day. These patients showed an improvement in echocardiographic findings after more than one year of tranilast administration, suggesting that inhibition of TRPV2 might become a new therapeutic target for heart failure and myopathy. In the following single-arm, open-label, multicenter study, 34 MD patients with advanced heart failure were enrolled; of which, 17 were in the full analysis set (FAS) [10]. Tranilast as an additional therapy was administered orally for 28 weeks at a dose of 300 mg/day, divided into three daily doses. In contrast to the pilot study, no obvious improvement in cardiac function was observed owing to the short-term administration period; however, tranilast may have the potential to prevent the progression of cardiac dysfunction and reduce cardiac events and mortality [11]. In this trial, we also collected both biochemical and urinary test data from the participants for an exploratory study. Furthermore, we performed a urinary metabolomics study focused on oxidative fatty acids, that is, bioactive lipids derived from polyunsaturated fatty acids (PUFA). Prostaglandin (PG)-mediated inflammation is involved in the pathology of MD, and urinary levels of tetranor-PGDM (tetranor-prostaglandin D metabolite), a metabolite of PGD_2_, are a useful marker of pathological progression [12,13]. In this study, we report the alteration of urinary metabolomic levels before and after tranilast administration and their correlation with other markers, such as oxidative stress markers. This study aimed to evaluate whether these metabolites have the potential to be used as biomarkers to assess improved cardiac function or prevention of the progression in MD.

## 2. Results

### 2.1. Alteration of Urinary Metabolites in a Long-Term Study

Of the 17 patients in the FAS, urine samples at 0, 4, 12, and 24 weeks after tranilast administration were available only for six (long-term study, Table 1). The reason why small numbers of samples were collected was that urinary metabolomic studies were exploratory, accompanied by clinical studies, and urine collection was not mandatory in participating institutes. Although we measured 88 target metabolites using selective reaction monitoring mass spectrometry (SRM-MS), 24 metabolites were detected in none of the specimens, 37 metabolites were detected in less than half of the specimens, and 27 metabolites were detected in more than half of the specimens. The normalized peak areas and creatinine (Cre) concentrations are listed in Supplementary Table S1. Twelve metabolites were detected in all specimens (six patients, four time points), and we focused on these. Of the twelve metabolites, four were linoleic acid (LA) metabolites (13-HODE, 9-HODE, 13-OxoODE, and trans-EKODE), four were arachidonic acids and their metabolites (2,3-dinor-8-iso prostaglandin F2α, tetranor-PGAM, tetranor-PGDM, and tetranor-PGEM), two were eicosapentaenoic acid (EPA) and its metabolite (prostaglandin E_3_), and docosahexaenoic acid (DHA) (Supplementary Table S1). In addition, we focused on urinary 8-hydroxy-2′-deoxyguanosine (8-OHdG), a biomarker for oxidative damage to nucleic acids, which was measured in these six patients (Table 2).

**Figure 1:**
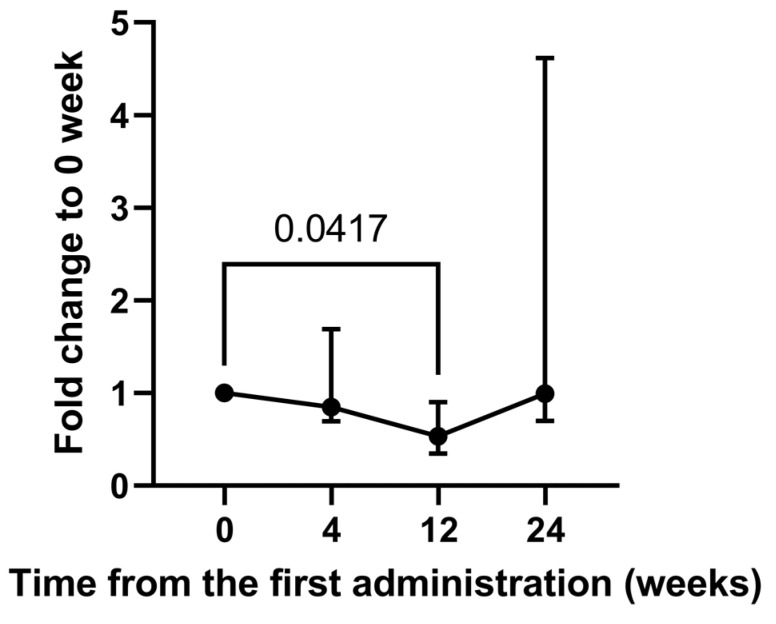
Alteration of urinary tetranor-PGDM level in MD patients between pre- and post-administration of tranilast. Fold-changes were calculated as ratios of levels at 4, 12, and 24 weeks relative to 0 weeks for each patient. Data were represented as medians with interquartile ranges of N = 6. *p*-values were determined using the Dunn’s post-hoc analysis.

To identify the metabolites associated with response to tranilast therapy, their urinary levels were compared before and after administration of tranilast (Table 2). Of the 13 metabolites including 8-OHdG, levels of tetranor-PGDM and tetranor-PGEM, major urinary metabolites of PGD_2_ and PGE_2_, respectively, changed significantly after administrations across four time points (*p* = 0.0292 and *p* = 0.0073 by Friedman’s test). However, post-hoc tests did not show any significant difference in tetranor-PGEM levels at 4, 12, and 24 weeks after tranilast administration compared with those of pre-administration. In contrast, urinary levels of tetranor-PGDM after 12 weeks of tranilast administration were significantly lower (0.53-fold) than those before tranilast administration (*p* = 0.0417 by post-hoc analysis, Figure 1).

**Figure 2:**
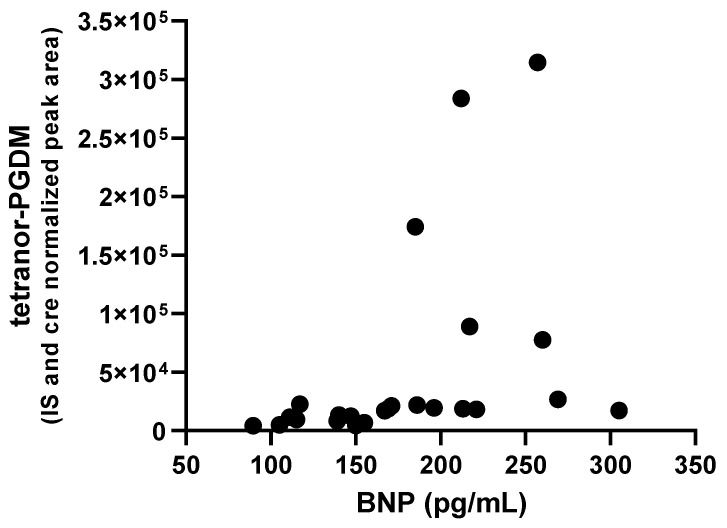
Correlation between BNP and urinary tetranor-PGDM level (r = 0.7052). Data on four time points of six patients were plotted.

Next, to examine whether the decrease in tetranor-PGDM is associated with the prevention of the progression of cardiac dysfunction, the correlation between BNP concentration and urinary levels of tetranor-PGDM was assessed in six patients at four time points. A positive correlation was observed, with a Spearman’s rank correlation coefficient r of 0.7052 (*p* = 0.0001), suggesting that urinary tetranor-PGDM might reflect cardiac function in our patients (Figure 2).

### 2.2. Alteration of Urinary Metabolites in a Short-Term Study

**Figure 3:**
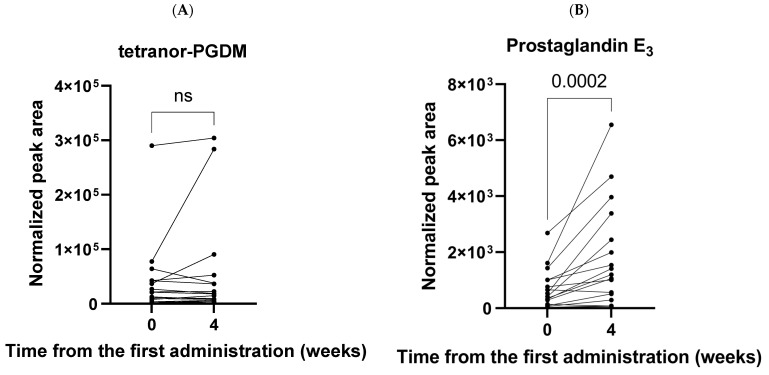
Comparison of normalized peak area of (**A**) tetranor-PGDM and (**B**) prostaglandin E_3_ in 17 DMD patients between pre- and post-administration. *p*-values were determined using the Wilcoxon signed-rank test. ns, not significant.

In the short-term study (Table 1), metabolome data, before and 4 weeks after tranilast administration, for 17 patients with FAS were compared using the Wilcoxon signed-rank test. There were no significant differences in the 12 metabolites, including tetranor-PGDM (Supplementary Table S2, Figure 3). This is consistent with a long-term study in which the tetranor-PGDM levels did not change after 4 weeks, but changed after 12 weeks of tranilast administration in six patients. In contrast, the urinary level of prostaglandin E_3_ (PGE_3_) was significantly increased 4 weeks after tranilast administration compared to before administration (*p* = 0.0002, Figure 3). Median fold changes in PGE_3_ levels at 4 weeks to pre-administration of tranilast was 3.15 (N = 17).

**Figure 4:**
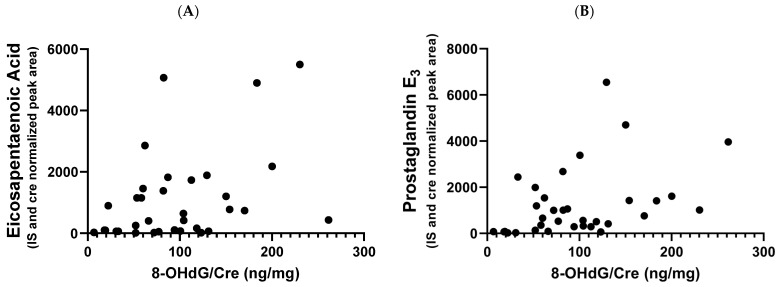
Correlation between 8-OHdG and urinary metabolite levels. (**A**) PGE_3_ level (r = 0.4625) and (**B**) EPA level (r = 0.4112). Data on two time points for 17 patients were plotted. 8-OHdG, 8-hydroxy-2′-deoxyguanosine levels, an oxidative stress marker.

The correlation of PGE_3_, which is derived from EPA, with the concentration of oxidative stress marker 8-OHdG, was examined because EPA may alter oxidative status and immune function [14]. There was a significant positive correlation between PGE_3_ and 8-OHdG, with a Spearman’s rank correlation coefficient r of 0.4625 (*p* = 0.0059) (Figure 4). EPA and 8-OHdG levels were weakly correlated (r = 0.4112, *p* = 0.0157).

## 3. Discussion

To the best of our knowledge, this is the first urinary metabolome analysis of MD patients with advanced cardiomyopathy intended to evaluate the effects of tranilast administration. In this clinical trial, TRPV2 expression on the mononuclear cell surface was reduced after administration of tranilast and cardiac biomarkers such as BNP remained stable [11], suggesting a protective effect of tranilast against the progression of heart failure. We previously reported that PGD_2_ in myocardial tissues from J2N-k hamsters was significantly increased in the symptomatic phase (16 weeks) but not in the presymptomatic phase (4 weeks), compared with that in J2N-n healthy control hamsters [15]. In a clinical study, urinary levels of tetranor-PGDM were shown to be increased in DMD patients compared with those in age-matched healthy control subjects and increased with advancing age [12]. These results suggest that PGD_2_-mediated inflammation plays a key role in myocardial damage in the progression of MD. An increase in urinary tetranor-PGDM levels might help explain the progression and symptomatic presentations, such as ambulatory difficulty, associated with DMD [13]. Therefore, we investigated whether tranilast administration suppressed the increase in urinary tetranor-PGDM levels. This study aimed to explore urinary biomarkers, including tetranor-PGDM, to assess improved cardiac function or prevention of the progression of MD.

We demonstrated that urinary tetranor-PGDM levels decreased after 12 weeks of tranilast administration in a long-term study. Urinary tetranor-PGDM levels at 4 and 24 weeks did not increase but were stable, that is, 0.85 and 0.99-fold before tranilast administration, respectively. In agreement with the long-term study, urinary levels of tetranor-PGDM at 4 weeks after tranilast administration did not increase (1.03-fold) compared with the levels before tranilast administration in 17 patients in the short-term study. Tetranor-PGDM has been shown to be a major urinary metabolite of PGD_2_ and reflects its biosynthesis [16]. PGD_2_ is a cyclooxygenase (COX) product of arachidonic acid that activates D-prostanoid receptors to modulate vascular, platelet, and leukocyte functions in vitro. PGD_2_ is implicated in both the development and resolution of inflammation, and its synthetic enzyme, hematopoietic-type prostaglandin D synthase was reported to be expressed in necrotic muscle fibers of DMD patients [17]. This indicates that the inflammatory mediator PGD_2_ plays a role in DMD pathology. Tranilast suppresses inducible cyclooxygenase-2 (COX-2) expression [18,19], resulting in decreased PGD_2_ synthesis [20]. Furthermore, tranilast inhibits the production of PGD_2_ by inhibiting PGD synthetase [21]. Considering that COX-2 expression is induced in necrotic and fibrotic lesions in mdx mice [22], the fact that tetranor-PGDM was stable at 4 and 24 weeks and decreased 12 weeks after tranilast administration suggests that PGD_2_-related MD progression may have been prevented by tranilast administration. To assess whether the effects of tranilast are mediated by the inhibition of TRPV2, it is necessary to examine the effects of an inhibitory antibody specific to TRPV2 on urinary tetranor-PGDM levels [23]. In a pilot study, favorable changes in echocardiographic findings were observed in two MD patients with advanced cardiomyopathy after more than one year of tranilast administration [3]. Four weeks may not be sufficient to assess the effect of tranilast on cardiac function. After 24 weeks of tranilast administration, only half of the six patients had lower levels of tetranor-PGDM than those before tranilast administration. The participants in this study had severe heart failure and poor health conditions compared with those in the pilot study.

There was a significant correlation between urinary tetranor-PGDM levels and BNP concentration (*p* = 0.0001, Spearman rank correlation test), indicating that tetranor-PGDM correlates with cardiac function that can be assessed using BNP (Figure 2). This result suggests that tetranor-PGDM may be a useful biomarker for heart failure, similar to conventional BNP levels. It remains unclear whether tetranor-PGDM is more sensitive than BNP as a biomarker for early-stage heart failure in MD patients, because this study was limited to patients whose BNP levels were >100 pg/mL during standard administration. It is necessary to evaluate the correlation between tetranor-PGDM and BNP in earlier patients with various stages of heart failure to assess its potency as a biomarker of cardiac function.

This study was the first report of a significant increase in PGE_3_ after 4 weeks of tranilast administration (*p* = 0.0002) (Figure 3). Tetranor-PGEM levels were also slightly increased (1.18-fold) after 4 weeks of tranilast administration with borderline significance (*p* = 0.0507). Although the mechanisms underlying the increase in PGE_3_ following tranilast administration are unclear, it is possible that the shunting effects after PGD synthase inhibition by tranilast lead the increments of PGE_3_. PGE_3_, which shares a metabolic pathway with PGE_2_, can be produced by nearly all cell types in the heart, such as fibroblasts, myocardial cells, and vascular endothelial cells and infiltrating inflammatory cells [24]. An increase in PGE_3_ might have a favorable effect on cardiac function. A high intake of EPA causes increased production of anti-inflammatory eicosanoids such as PGE_3_ [25,26] and exerts cardioprotective effects in coronary artery disease and sudden cardiac death [27,28,29,30]. In this study, patients with MD did not have any dietary restrictions or supplementation with n-3 PUFA. Indeed, urinary EPA levels did not increase after tranilast administration (Supplementary Table S2). Therefore, it is unlikely that the increase in urinary PGE_3_ levels could be attributed to high EPA intake. PGE_3_ interacts with PGE receptors (e.g., EP1–4) together with PGE_2_, albeit with different binding affinities and potencies [31]. An increase in PGE_3_ production might suppress the pro-inflammatory effects of PGE_2_ at sites of inflammation [32,33]. In this long-term study, urinary levels of PGE_3_ at 4, 12, and 24 weeks were 1.87-, 0.91-, and 1.78-fold, respectively, of those at 0 weeks, whereas urinary levels of tetranor-PGEM, the major urinary metabolite of PGE_2_, at 4, 12, and 24 weeks were 1.36-, 0.52-, and 1.90-fold, respectively, of those at 0 weeks; however, these changes were not significant due to individual variation (Table 2). This data suggests that alterations in the urinary levels of tetranor-PGEM and PGE_3_ may be synchronized. Tranilast may be effective in regulating the balance between pro-inflammatory and anti-inflammatory responses in patients with MD.

Oxidative stress is involved in the pathogenesis of dilated cardiomyopathy (DCM). We previously demonstrated a mild reduction in glutathione levels and a compensatory increase in ophthalmate levels in the cardiac tissue of 16-week-old J2N-k hamsters, suggesting increased oxidative stress [15]. Patients with DCM exhibit increased plasma glutathione levels and lipid peroxidation products such as malondialdehyde [34], and total plasma peroxide levels are inversely correlated with cardiac ejection fraction [35]. There are no reports on the effect of PGE_3_ on oxidative stress, but EPA has been suggested to have antioxidant properties [36,37]. High PGE_3_ levels may have a protective effect against cardiomyopathy in MD patients with high oxidative stress; however, the underlying mechanisms are unknown.

In conclusion, this study demonstrated that urinary levels of tetranor-PGDM were significantly decreased by tranilast administration and were correlated with BNP, a conventional biomarker. Tranilast suppresses PG-mediated inflammation and prevents cardiac dysfunction progression. Examining urinary tetranor-PGDM levels helps with understanding the extent of advanced heart failure in patients with MD. Our results also suggest that PGE_3_ that is increased by tranilast administration at earlier stages may be a novel biomarker with therapeutic effects against heart failure in patients with MD. However, this is an exploratory study, and further validation is needed to determine whether these metabolites are potential biomarkers of improved cardiac function or prevention of MD progression.

## 4. Materials and Methods

### 4.1. Chemicals and Reagents

LC-MS-grade water was purchased from Kanto Chemical Co., Inc. (Tokyo, Japan). Formic acid, methanol (for LC-MS), acetonitrile (for LC-MS), and isopropanol (for LC-MS) were purchased from FUJIFILM Wako Pure Chemical Co., Ltd. (Osaka, Japan). Authentic standards for oxidative fatty acids were obtained from Cayman Chemicals (Ann Arbor, MI, USA) or Santa Cruz Biotechnology (Santa Cruz, CA, USA).

### 4.2. Patients and Collection of Urine Samples

A total of 17 patients with MD with advanced heart failure, corresponding to FAS in the clinical trial, were the subjects of a urinary metabolomic study. Among the MD patients, 11 were classified as having DMD, three as having Becker muscular dystrophy, and one as having limb girdle muscular dystrophy. Patient characteristics are summarized in Table 1. The 24 h urine samples were collected from 11 patients at all time points, while spot urine was collected at either time point in six patients (Supplementary Table S1). Samples were then transferred into an Eppendorf tube, which was stored at −80 °C until use. Urinary 8-OHdG concentrations were determined using an ELISA kit by outsourcing contractors, SRL, Inc. (Tokyo, Japan).

### 4.3. Urine Sample Preparation for MS Analysis

Samples were thawed in an ice bath to prevent sample degradation and centrifuged (13,200× *g*) at 4 °C for 3 min. Next, 200 μL of the supernatant was added to 2750 μL of 25% ACN in a glass tube and mixed thoroughly. Then, 50 μL of deuterated internal standard solution (160 nM each of prostaglandin E_2_-d4, leukotriene B_4_-d4, 15(S)-HETE-d8, prostaglandin F_2α_-d9) was added to a glass tube, and the samples were subsequently vortexed for 5 min. Oxidative fatty acids were extracted according to earlier published protocols with some modifications [38,39,40]. In brief, an Oasis MAX SPE Cartridge (Waters, Milford, MA, USA) was conditioned with 3 mL of ACN, followed by 3 mL of 25% ACN. The entire pre-treated sample was loaded onto the Oasis MAX SPE Cartridge and then washed with 3 mL of 25% ACN and 3 mL of ACN. The samples were eluted with 2.0 mL of 1% formic acid in ACN and collected in a glass amber bottle. Finally, the eluted samples were evaporated under nitrogen and reconstituted in 500 μL of 50/50 MeOH/ACN. For SRM-MS analyses, 225 μL of extracted samples was dried in a SpeedVac (Thermo Fisher, Paisley, UK) and resuspended in 30 μL of loading buffer (60% MeOH).

### 4.4. SRM-MS Analysis

The analyses were performed on an LCMS-8050 Triple Quadrupole Liquid Chromatograph Mass Spectrometer (Shimadzu, Kyoto, Japan) equipped with a heated ESI source and a Shimadzu LC system, which consisted of Shimadzu LC-20AD pumps and an SIL-30AC autosampler. Chromatographic separations were carried out using a Kinetex C8 column (150 mm × 2.1 mm, 2.6 μm) (Phenomenex, Torrance, CA, USA) at a flow rate of 0.3 mL/min. The mobile phases consisted of acetonitrile/methanol (75:25, *v*:*v*) (solvent A) and 0.1% formic acid in water (solvent B), and a gradient elution program was set as follows: 0–7 min, 90–75% B; 7–14 min, 75–65% B; 14–27 min, 65–25% B; 27–27.1 min, 25–5% B; 27.1–34 min, 5% B; 34–34.1 min, 5–90% B; 34.1–45 min, 90% B. The column oven temperature was 40 °C. The injection volume was 15 µL. The ESI-MS conditions were as follows: nebulizer gas flow, 3 L/min; heating gas flow, 10 L/min; drying gas flow, 10 L/min; heat block temperature, 400 °C; DL temperature, 250 °C; and spray voltage, −3.5 kV for negative mode. The SRM transitions for each of the 88 oxidative fatty acids were obtained from our previous studies [15,41]. Parameters such as Q1 pre-rod bias voltage, CE, Q3 pre-rod bias voltage, and retention time (RT) were determined through optimization using standard materials, and the scheduled-SRM method was constructed with fixed RT window widths (3 min) [42].

### 4.5. Data Analysis and Statistical Analysis

The creatinine concentration of each urine specimen was measured using a creatinine (urinary) Colorimetric Assay Kit (Cayman Chemicals) according to the manufacturer’s protocol. Data from SRM-MS were analyzed using LabSolutions version 5.91 (Shimadzu), and the integrated peak area of each metabolite was normalized to the IS and creatinine response. The normalized peak areas were statistically compared between pre- and post-tranilast administration using Prism 9 (GraphPad Software, San Diego, CA, USA), with statistical significance set at *p* < 0.05. Analysis was performed on 6 patients with urine specimens at 0, 4, 12, and 24 weeks (long-term study) and 17 patients with urine specimens only at 0 and 4 weeks (short-term study). For the long-term study, statistical significance was determined using Friedman’s test with Dunn’s post-hoc analysis to examine differences in metabolite levels pre- and post-tranilast administration. Correlations between urinary metabolite levels and other markers, such as BNP concentration and 8-OHdG, were evaluated using Spearman’s rank correlation coefficient. For the short-term study, the Wilcoxon signed-rank test was used to calculate statistical significance.

## Figures and Tables

**Table 1:** Patient characteristics in each study.

Study		Long-Term Study	Short-Term Study
No. of patients *		6	17
Urine collection point (weeks)		0, 4, 12, 24	0, 4
Age (mean ± SD)		28.5 ± 1.4	33.1 ± 7.5
Sex	male/female	6/0	16/1
Diseases	Duchenne muscular dystrophy	5	11
	Becher muscular dystrophy	1	3
	Limb-Girdle muscular dystrophy	0	1
BNP (pg/mL) (GM ± GSD)	0 weeks	182.0 ± 1.4	192.9 ± 1.7
	4 weeks	162.9 ± 1.4	184.7 ± 1.8
	12 weeks	169.0 ± 1.5	-
	24 weeks	168.3 ± 1.4	-

* Patients in the long-term study were also included in short-term-study patients’ group.

**Table 2:** Median fold changes in metabolite levels after tranilast administration.

	Median Fold Change			*p*-Value
	4W/0W	12W/0W	24W/0W	
13-HODE	0.91	0.59	0.70	0.6093
9-HODE	1.46	1.39	1.35	0.5120
13-OxoODE	0.90	1.27	0.49	0.8438
2,3-dinor-8-iso-Prostaglandin F_2α_	0.68	0.50	1.31	0.1555
Arachidonic Acid	0.50	0.54	1.09	0.6787
Docosahexaenoic Acid	0.31	0.51	1.05	0.6787
Eicosapentaenoic Acid	0.68	1.10	1.16	0.1555
Prostaglandin E_3_	1.87	0.91	1.78	0.1633
tetranor-PGAM	0.92	0.63	1.09	0.1081
tetranor-PGDM	0.85	0.53	0.99	0.0292
tetranor-PGEM	1.36	0.52	1.90	0.0073
trans-EKODE-(E)-Ib	0.21	0.30	0.51	0.3855
8-OHdG	1.01	1.02	0.99	>0.9999

Statistical analysis was performed using Friedman’s test to detect differences in metabolite levels from 0 weeks to 24 weeks. *p*-values < 0.05 were considered statistically significant.

## Data Availability

All the data generated in this study are present in the main manuscript and Supplementary Files.

## Supplementary Materials

The following supporting information can be downloaded at: https://www.mdpi.com/article/10.3390/ijms24032167/s1.
